# IR Sensor Synchronizing Active Shutter Glasses for 3D HDTV with Flexible Liquid Crystal Lenses

**DOI:** 10.3390/s131216583

**Published:** 2013-12-03

**Authors:** Jeong In Han

**Affiliations:** Department of Chemical and Biochemical Engineering, Dongguk University-Seoul, Seoul 100-715, Korea; E-Mail: hanji@dongguk.edu; Tel.: +82-2-2260-3364; Fax: +82-2-2268-8729

**Keywords:** IR sensor synchronizing, active shutter goggle, 3D HDTV, flexible liquid crystal lens

## Abstract

IR sensor synchronizing active shutter glasses for three-dimensional high definition television (3D HDTV) were developed using a flexible liquid crystal (FLC) lens. The FLC lens was made on a polycarbonate (PC) substrate using conventional liquid crystal display (LCD) processes. The flexible liquid crystal lens displayed a maximum transmission of 32% and total response time of 2.56 ms. The transmittance, the contrast ratio and the response time of the flexible liquid crystal lens were superior to those of glass liquid crystal lenses. Microcontroller unit and drivers were developed as part of a reception module with power supply for the IR sensor synchronizing active shutter glasses with the flexible liquid crystal lens prototypes. IR sensor synchronizing active shutter glasses for 3D HDTV with flexible liquid crystal lenses produced excellent 3D images viewing characteristics.

## 1. Introduction

The success of three-dimensional (3D) movies such as Avatar has spurred great interest in the technology, and has increased the commercial potential of 3D high definition television (HDTV). Among the available modes of 3D HDTV, the type utilizing active shutter glasses is most favored because of the excellent image quality. Typically, IR sensor synchronizing active shutter type 3D HDTV displays stereoscopic 3D images, presenting the image through the left eye while blocking the view through the right eye. Subsequently, the pattern is reversed. Repeat of this cycle at a fast repetition rate eliminates interference with the perceived fusion of the two images into a single 3D image.

IR sensor synchronizing active shutter glasses include a twisted nematic (TN) liquid crystal lens, optical transmitter, and electronics that include IR sensors, microcontroller unit (MCU), driver, and control circuits. Among these parts, the TN liquid crystal lens operates as an optical shutter. Each eye glass of the active shutter glasses contains a TN liquid crystal layer, which becomes dark when the voltage is applied and transparent in the absence of applied voltage. Control of the glasses can be achieved by an alternative trigger signal that allows sequential darkening of one eye and then the other in synchrony with the refresh rate of the screen through IR sensor. The synchronization of the trigger signal to video equipment is realized wirelessly by an infrared radio frequency or Bluetooth optical transmitter [1,2].

Our research group was the first to report the applications of flexible liquid crystal lenses to the active shutter glasses [1] based on some previous suggestions by the Turner group [2] for HDTV systems using active optical shutter glasses and the application of the TN liquid crystal lens device to active shutter glasses for HDTV.

TN liquid crystal lenses have been fabricated with rigid and brittle glass substrates. They are easily damaged or broken by external forces or blunt impacts, which can cause serious, even fatal, eye wounds. Moreover, glass materials are too brittle or hard to control, which has limitations in applications requiring various shapes or designs of the lenses, such as flexible and curvilinear devices. To overcome these problems, proposed flexible displays or lens systems involve replacement of the rigid glass substrate by a flexible polymeric substrate. The liquid crystal lens can be thought of as a one pixel flexible liquid crystal display. It would be more easily designed and fabricated than the flexible liquid crystal display because it has only one pixel. Therefore, IR sensor synchronizing active shutter glasses including polymeric liquid crystal lenses are required to improve the endurance and the flexibility of the system.

In this paper, the development of a one pixel flexible liquid crystal lens system as well as the implemented electronics including IR sensor, MCU, driving circuits, and controllers for use in robust and flexible active shutter glasses is presented. The transmittance, power consumption, and response time of the proposed shutter glasses are measured as a function of applied bias, which are comparable or outperform those based on conventional glass substrates.

## 2. Experimental Section, Results and Discussion

### 2.1. Construction and Measurement of the Flexible LC Lens

**Figure 1.**
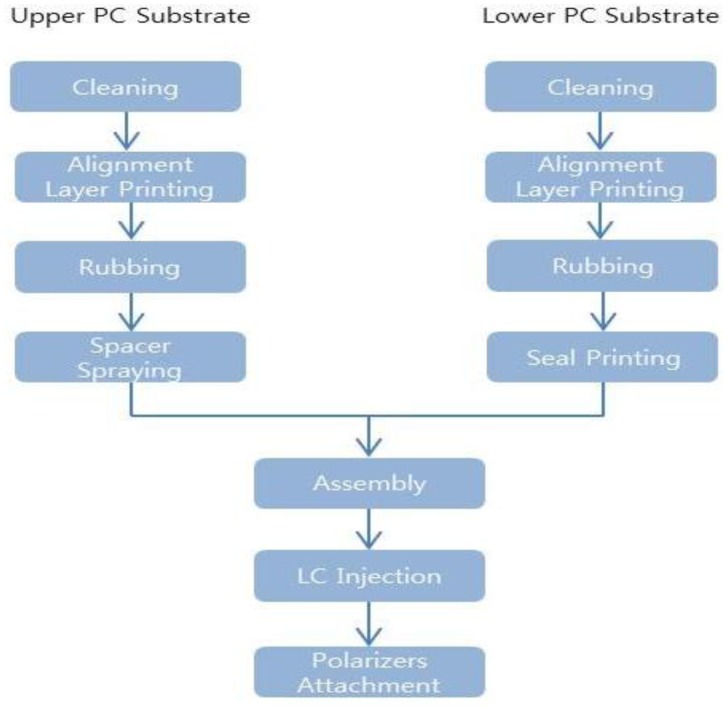
Flow chart of the fabrication process for the flexible liquid crystal lens.

A flexible liquid crystal lens consists of a flexible LCD with one huge pixel. Its fabrication flow chart is shown in Figure 1. Conventional fabricating procedures are used for the flexible LCD [3–6], with the exception of the pixel patterning process. Because the device structure of the flexible liquid crystal lens is exactly same to the glass based liquid crystal display with one pixel, the fabrication processes of the flexible liquid crystal lens are almost identical to the conventional fabricating processes without no patterning. However, because of very low thermal stability of the flexible substrate, lots of low temperature processes and materials have to be introduced for the realization of the flexible devices as described in the previous papers on the low temperature processes [3–6] and materials such as polymer substrate [3–6], metal [5], insulator [5], rubbing, color filters [6] and so on for their low temperature processes. The TN mode for the flexible liquid crystal lens was made to enable use of the high speed liquid crystal for the HDTV. The flexible TN liquid crystal lens was fabricated on a substrate of polycarbonate (PC) film of 130 m onto which the transparent conducting layer of indium tin oxide (ITO) film was deposited by sputtering. After cleaning, a polyimide (PI) rubbing was performed on the ITO films of the PC substrates for the liquid crystal alignment between the ITO-coated PC films. The sheet resistance of ITO film was 16.81 Ω. The PI rubbing direction of the upper and lower PC films was 90° to twist the liquid crystal layer. The spacers were then splayed on one PC film and sealant was dispensed on the other PC substrate. The upper and lower PC films were assembled and the liquid crystal was injected to the flexible TN liquid crystal device. Finally, polarizers were attached to make very large one pixel flexible liquid crystal lens.

**Figure 2.**
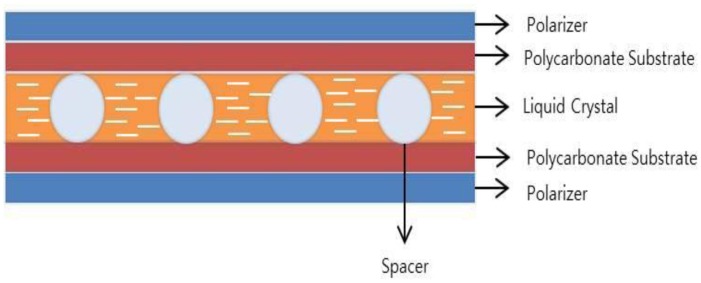
Vertical view of the flexible liquid crystal lens.

Figure 2 shows a schematic of the vertical view of the fabricated flexible liquid crystal lens. Polarizers were placed at the outmost layers of the liquid crystal lens on the PC substrate. The liquid crystal layer and the spacers between the PC films are indicated. The cell gap was 5 µm.

**Figure 3.**
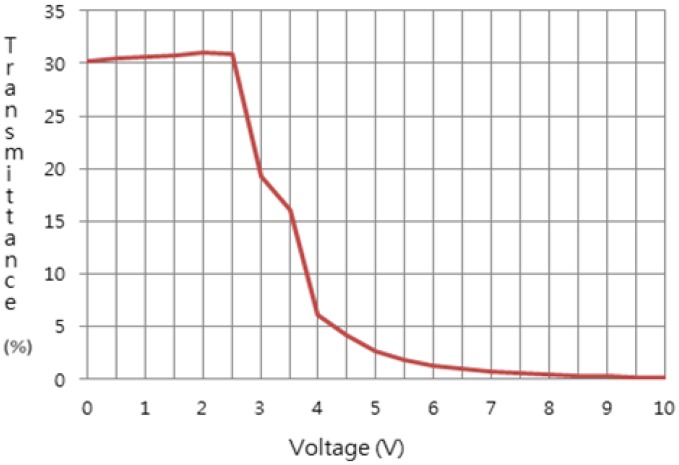
Light transmittance with the applied voltage of the flexible liquid crystal lens fabricated on PC substrate.

The light transmittance with the applied voltage and the response time of the flexible liquid crystal lens were measured. The light transmittance obtained with the applied voltage is shown in Figure 3. In the absence of applied voltage, light passed through the liquid crystal layer. The maximum transmittance of the flexible TN liquid crystal lens was 32% and that of the glass super TN (STN) liquid crystal lens was less than 35%. The difference of liquid crystal mode translated to a slightly smaller transmittance of the flexible TN liquid crystal lens compared to the glass STN liquid crystal lens. This overall low transmittance was due to the light absorption of the polarizers attached to the upper and lower PC films. However, some commercial products showed a much lower transmittance of 23% [7].

The contrast ratio (C/R) of 177:1 was calculated (Figure 3). Commercial active shutter glasses with glass STN liquid crystal lens typically display a ratio of 100:1, which reflects a slower response time than that of flexible TN liquid crystal lens. Data of commercial active glasses were quoted from the active shutter glasses database of the 3D@Home Consortium [7].

**Figure 4.**
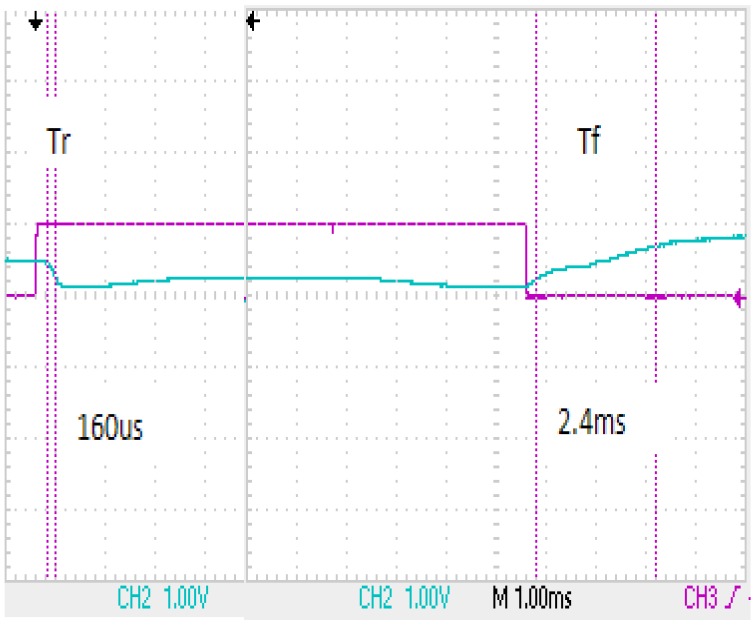
Rising and falling response time of the flexible liquid crystal lens.

The response time of the flexible liquid crystal lens was measured (Figure 4). The rising response time and falling response time were 160 µs and 2.4 ms, respectively. The total response time was 2.56 ms. These response times were markedly faster than those of the thin film transistor (TFT) LCD. The presently developed lens was only one pixel, contrasting with the millions of pixels in the TFT LCD. Like the transmittance and contrast ratio of glass TN and STN LCD, the flexible liquid crystal lens displayed a much faster response time than the glass TN (354 ms) and STN liquid crystal lens (3 ms). Especially, the response time of the flexible liquid crystal lens was markedly faster than that of the commercial liquid crystal lens.

Table 1 summarizes comparative data of the presently developed flexible liquid crystal lens and the general commercial glass liquid crystal lens for the active shutter glasses. Compared to the commercial glass liquid crystal lens, the flexible liquid crystal lens showed excellent electro-optical properties.

**Figure 5.**
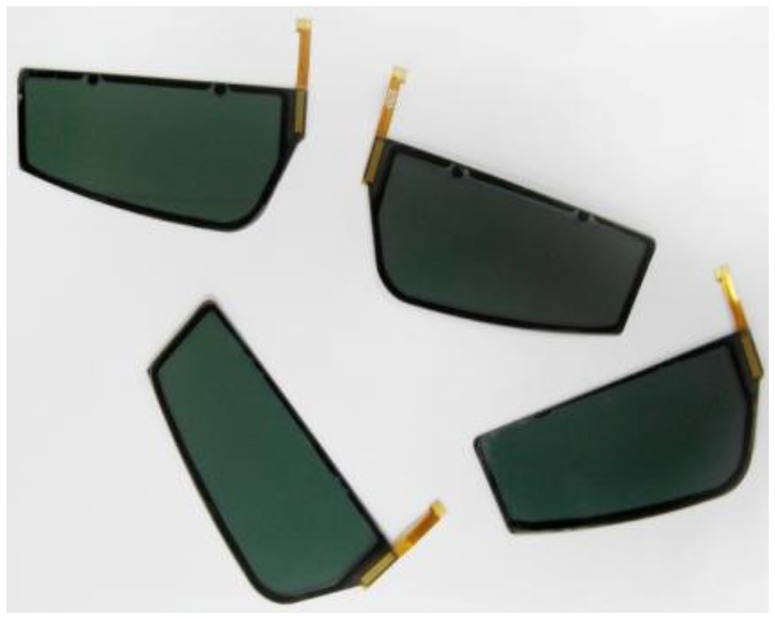
Examples of the fabricated flexible liquid crystal lens.

A photograph of the flexible liquid crystal lens fabricated on PC substrate is shown in Figure 5. The non-rectangular shape was advantageous for the flexible liquid crystal lens. Although the transmittance was not high, the light passed through the flexible liquid crystal lens. The slight darkness was caused by the polarizer attached to the surface of the lens.

### 2.2. Construction of the Prototype Active Shutter Glasses with the Flexible Liquid Crystal Lens and Measurements of the Electro-Optical Properties

**Figure 6.**
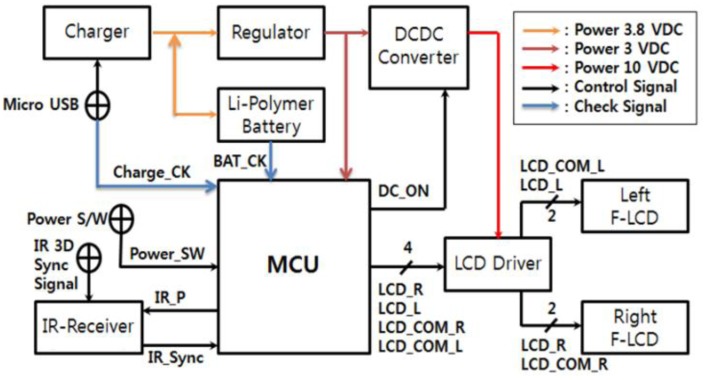
Block diagram for the active shutter glasses with the flexible liquid crystal lens.

A block diagram of the reception module for the active shutter glasses with the flexible liquid crystal lens is shown in Figure 6. As a power supply, the charger, the regulator and the DC-DC converter were added for the charging of the lithium(Li)-polymer battery. It supplies a constant voltage output of 3 VDC and converts 3 VDC to 10 VDC for driving the flexible liquid crystal lens. To control the right and left flexible liquid crystal lens with the synchronization with the infrared (IR) 3D signal, a commercial MCU was used. LCD driver IC was adapted to drive the flexible liquid crystal lens. The power source was a Li-polymer battery. In upper right corner of Figure 6, power line of 3.8 VDC, 3 VDC and 10 VDC and signal line of control and check are illustrated. To make a circuit board for the reception module for the active shutter glasses with the flexible liquid crystal lens, the block diagram were realized using detailed circuits for IR sensor, Li battery, regulator, DC-DC converter, MCU, LCD driver, power switch, battery, and charge check.

**Figure 7.**
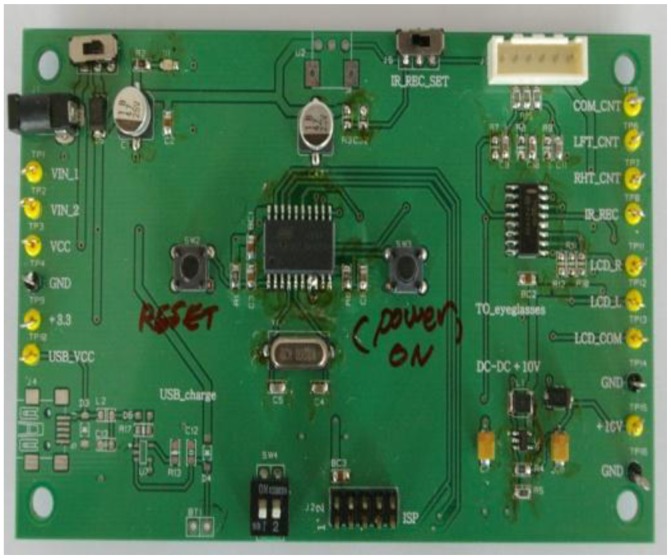
Test board of the reception module for the active shutter glasses with the flexible liquid crystal lens.

**Figure 8.**
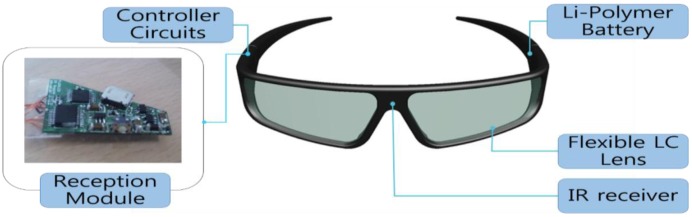
The prototype of the active shutter glasses with the flexible liquid crystal lens.

Figure 7 displays the fabricated driving board of the reception module for the active shutter glasses with the flexible liquid crystal lens. After several tests and revisions of block diagrams and circuit boards, the finally revised printed circuit board module of Figure 7 is used for the prototypes of the active shutter glasses with the flexible liquid crystal lens. The prototype of IR sensor synchronizing active shutter glasses with the flexible liquid crystal lens is shown in Figure 8. Reception module, controller circuit, Li-polymer battery, IR receiver, and flexible liquid crystal lens can be identified in Figure 8.

The prototype of IR sensor synchronizing active shutter glasses with the flexible liquid crystal lens weighs 35 g. The low power consumption (2.3 mA) and the 75 mAh Li-polymer battery allow a 30 h continuous use of the active shutter glasses with the flexible liquid crystal lens. Also, electromagnetic susceptibility (EMS) and electromagnetic interference (EMI) test results indicated the great stability of the active shutter glasses with the flexible liquid crystal lens.

IR sensor synchronizing active shutter glasses for 3D HDTV with flexible liquid crystal lens showed excellent viewing characteristics for 3D image quality. Also, the flexible liquid crystal lens of the prototype of the active shutter glasses appears slightly curve-shape. If the glass substrate is used for the substrate of the lens, it can be curved and achieved only when the flexible liquid crystal lens is adopted. Although the degree of bending is very small, it will be more improved further.

**Figure 9.**
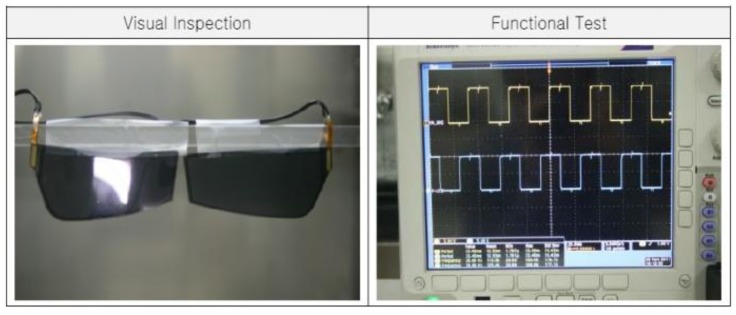
Results of low-high temperature operating test.

Low-high temperature operating test results of the active shutter glasses with the flexible liquid crystal lens are summarized in Figure 9. The low-high temperature operating test was carried out by several thermal cycles from 0 °C to 40 °C for 8 h. No damage occurred and the operation of the active shutter glasses with the flexible liquid crystal lens was superior.

## 3. Conclusions

A flexible liquid crystal lens for IR sensor synchronizing active shutter glasses for 3D HDTV is developed. The flexible liquid crystal lens of TN mode is made on a PC substrate through conventional processes. The flexible liquid crystal lens showed a maximum transmittance of 32% and a total response time of 2.56 ms. The C/R ratio was 177:1, which was much higher and faster than that of the commercial glass liquid crystal lenses.

The prototype of IR sensor synchronizing active shutter glasses with the flexible liquid crystal lens required development of a reception module with a power supply, IR sensor, MCU, and drivers. The design of the active shutter glasses included a slightly curved flexible liquid crystal lens.

The IR sensor synchronizing active shutter glasses for 3D HDTV with flexible liquid crystal lenses showed excellent viewing properties for the 3D images of 3D HDTV. IR sensor synchronizing active shutter glasses with flexible liquid crystal lenses showed good stability during EMS tests and low-high temperature operation tests.

## Figures and Tables

**Table 1.** Comparison the flexible liquid crystal lens (TN mode) and the commercial glass liquid crystal lens (TN and STN mode) for the active shutter glasses.

**Mode**	Weight*(g)	**Transmittance (%)**	**Contrast Ratio (C/R)**	**Response Time (ms)**
Flexible LC Lens (TN)	35	TN : 32	TN: 177:1	TN: 2.56
Glass LC Lens (TN&STN)	50–90	TN : 23 STN : 30–40	TN : - STN: > 100:1	TN : 354–356 STN : 1.7–2.5

* The weight of the active shutter glasses with the flexible or glass liquid crystal.

## References

[1] Han J.I. Active Shutter Glasses for 3D HDTV with Flexible Liquid Crystal Lens.

[2] Turner T.L., Hellbaum R.F. (1986). LC shutter glasses provide 3-D display for simulated flight. Inform. Display.

[3] Kim Y.H., Park S.K., Moon D.G., Kim W.K., Han J.I. (2004). Organic thin film transistor-driven liquid crystal displays on flexible polymer substrate. Jpn. J. Appl. Phys..

[4] Kim Y.-H., Park S.-K., Moon D.-G., Kim W.-K., Han J.-I. (2004). Active-matrix liquid crystal display using solution-based organic thin film transistors on plastic substrates. Displays.

[5] Hong S.J., Lee C.J., Han J.I., Kim W.K., Moon D.G., Kwak M.G., Park S.K., Kim Y.H. (2002). Flexible metal-insulator-metal (MIM) devices for plastic film AM-LCD. Curr. Appl. Phys..

[6] Park S.K., Han J.I., Kim W.K., Kwak M.G., Hong S.J.C., Lee J., Min K.S., Park C.Y. (2001). Novel reflective color STN plastic film LCD with high brightness and parallax free image. SID Symposium Digest Tech. Papers.

[7] 3D Active Shutter Glasses Database. http://www.3dathome.org/files/ST4-04_Active_Shutter_Glasses_Database.pdf.

